# Advanced Glycation End Products Acutely Impair Ca^2+^ Signaling in Bovine Aortic Endothelial Cells

**DOI:** 10.3389/fphys.2013.00038

**Published:** 2013-03-11

**Authors:** Nadim Naser, Andrzej S. Januszewski, Bronwyn E. Brown, Alicia J. Jenkins, Michael A. Hill, Timothy V. Murphy

**Affiliations:** ^1^Department of Physiology, School of Medical Sciences, University of New South WalesSydney, NSW, Australia; ^2^Department of Medicine, St. Vincent’s Hospital, University of MelbourneFitzroy, VIC, Australia; ^3^Heart Research InstituteSydney, NSW, Australia; ^4^Department of Medical Pharmacology and Physiology, Dalton Cardiovascular Research Center, University of MissouriColumbia, MO, USA

**Keywords:** calcium signaling, endothelium, advanced glycation end products, reactive oxygen species

## Abstract

Post-translational modification of proteins in diabetes, including formation of advanced glycation end products (AGEs) are believed to contribute to vascular dysfunction and disease. Impaired function of the endothelium is an early indicator of vascular dysfunction in diabetes and as many endothelial cell processes are dependent upon intracellular [Ca^2+^] and Ca^2+^ signaling, the aim of this study was to examine the acute effects of AGEs on Ca^2+^ signaling in bovine aortic endothelial cells (BAEC). Ca^2+^ signaling was studied using the fluorescent indicator dye Fura-2-AM. AGEs were generated by incubating bovine serum albumin with 0–250 mM glucose or glucose-6-phosphate for 0–120 days at 37°C. Under all conditions, the main AGE species generated was carboxymethyl lysine (CML) as assayed using both gas-liquid chromatograph-mass spectroscopy and high-performance liquid chromatography. In Ca^2+^-replete solution, exposure of BAEC to AGEs for 5 min caused an elevation in basal [Ca^2+^] and attenuated the increase in intracellular [Ca^2+^] caused by ATP (100 μM). In the absence of extracellular Ca^2+^, exposure of BAEC to AGEs for 5 min caused an elevation in basal [Ca^2+^] and attenuated subsequent intracellular Ca^2+^ release caused by ATP, thapsigargin (0.1 μM), and ionomycin (3 μM), but AGEs did not affect extracellular Ca^2+^ entry induced by the re-addition of Ca^2+^ to the bathing solution in the presence of any of these agents. The anti-oxidant α-lipoic acid (2 μM) and NAD(P)H oxidase inhibitors apocynin (500 μM) and diphenyleneiodonium (1 μM) abolished these effects of AGEs on BAECs, as did the IP_3_ receptor antagonist xestospongin C (1 μM). In summary, AGEs caused an acute depletion of Ca^2+^ from the intracellular store in BAECs, such that the Ca^2+^ signal stimulated by the subsequent application other agents acting upon this store is reduced. The mechanism may involve generation of reactive oxygen species from NAD(P)H oxidase and possible activation of the IP_3_ receptor.

## Introduction

One of the earliest signs of vascular endothelial dysfunction observed in diabetes is impairment of endothelium-dependent vasodilatation. Impaired vasodilatation has been demonstrated in a number of experimental, animal models of hyperglycemia and diabetes (Tesfamariam et al., 1991; Fukao et al., 1997; De Vriese et al., 2000; Young et al., 2008; Ding and Triggle, 2010) as well as in clinical studies of both type 1 and type 2 diabetes subjects (McVeigh et al., 1992; Clarkson et al., 1996; Ting et al., 1996). While high blood glucose levels alone may have an inhibitory effect on endothelium-dependent vasodilatation (Creager et al., 2003; Gerich, 2003; Endemann and Schiffrin, 2004; Hartge et al., 2006), a further consequence of hyperglycemia is the enhanced formation of glycated species including advanced glycation end products (AGEs), a reaction between glucose/glucose metabolites and amine, guanidine and thiol groups on proteins, and other long-lived macromolecules. AGE formation is thought to contribute to many of the deleterious vascular effects of diabetes (Goldin et al., 2006; Negre-Salvayre et al., 2009) including impaired endothelium-dependent vasodilatation (Bucala et al., 1991; Rodriguez-Manas et al., 1993; Vallejo et al., 2000; Gao et al., 2008; Sena et al., 2012). AGEs may also damage the vasculature by cross-linking extracellular matrix and other vascular proteins, stiffening the vascular wall (Brownlee et al., 1986; Sims et al., 1996) as well as through binding to specific receptors for AGE (RAGE; Farmer and Kennedy, 2009; Yan et al., 2009), which subsequently generates inflammatory mediators including reactive oxygen species (ROS) and TNFα.

Formation of the key endothelium-derived vasodilator substance, nitric oxide (NO), and the activity of endothelium-derived hyperpolarizing factors (EDHFs) is dependent on an increase in endothelial cell [Ca^2+^]. Synthesis of NO from arginine by NO synthase (NOS) is a process that is dependent on several co-factors including Ca^2+^, the Ca^2+^-binding protein calmodulin, oxygen, tetrahydrobiopterin, and NAD(P)H (Moncada and Higgs, 2006). Although several candidate endothelium-derived hyperpolarizing (EDH) substances and mechanisms exist, Ca^2+^-activated K^+^ channels (K_Ca_) represent important common elements in their function. In endothelial cells, both intermediate- and small-conductance K_Ca_ (IK_Ca_ and SK_Ca_ respectively) are involved in most established EDH mechanisms (Feletou and Vanhoutte, 2009; Sandow et al., 2009). K_Ca_ require an increase in cytoplasmic [Ca^2+^] in order to be activated. In addition to its primary role in stimulating NOS and EDHF activity, and thus initiating endothelium-dependent vasodilatation, endothelial cell Ca^2+^ signaling is vital in regulating endothelial cell growth, proliferation, and angiogenesis (Munaron and Fiorio Pla, 2009) along with hemostasis and inflammatory responses (Hawkins et al., 2007; Sadler, 2009).

Vascular endothelial cell [Ca^2+^] may be altered by a wide variety of neural, humoral, and physical stimuli (Muller et al., 1999; Kwan et al., 2007; Sandow et al., 2012). Receptor-mediated increases generally involve activation of G_q/11_-protein and phospholipase C (PLC) and subsequent inositol 1,4,5-trisphosphate (IP_3_)-mediated release of intracellular Ca^2+^ stores (Sandow et al., 2012). The endoplasmic reticulum also possesses ryanodine receptors and Ca^2+^-induced Ca^2+^ release occurs in these cells. Extracellular Ca^2+^ entry is not through voltage-dependent Ca^2+^ channels as vascular endothelial cells typically do not express them. Ca^2+^ entry is sensitive to the electrochemical gradient across the membrane, such that Ca^2+^ entry is enhanced by hyperpolarization and reduced by depolarization of endothelial cells. Extracellular Ca^2+^ entry consists of a capacitive, store-operated component (capacitive Ca^2+^ entry or CCE), activated by depletion of intracellular stores, and/or entry through other mechanisms including receptor-operated channels and membrane Ca^2+^-exchangers. The mechanism of CCE into vascular endothelial cells is not fully understood and is somewhat controversial (Beech, 2009), with transient receptor potential (TRP) channels, the endoplasmic reticulum Ca^2+^ sensor STIM1, and the plasma-membrane Orai1 channel all implicated in the mechanism (Beech, 2009; Sandow et al., 2012). Receptor-operated channel mechanisms are primarily associated with the activity of various TRP-canonical (TRPC) and vanilloid (TRPV) subtypes (Sandow et al., 2012; Senadheera et al., 2012; Sonkusare et al., 2012).

Only a few studies have examined the direct effects of AGEs and other glycated species on endothelial cell Ca^2+^ signaling. In a previous study utilizing bovine aortic endothelial cells (BAEC) plated on glycated mixed matrix (EHS) protein, ATP-, and thapsigargin-induced increases in intracellular [Ca^2+^] were shown to be inhibited (Bishara et al., 2002a), suggesting glycated proteins can interfere with Ca^2+^ signaling in the endothelium. However the role of AGEs in, and the time-course of, this response (cells were plated on modified EHS for approximately 16 h) were not examined. Furthermore, while endothelial cell viability was significantly reduced by the glycated EHS matrix proteins it was noticeable that ATP-induced Ca^2+^ signaling was not impaired in cells similarly exposed to glycated fibronectin. Interestingly, the glycated fibronectin did not markedly inhibit cell viability (Bishara et al., 2002a), suggesting toxicity may have contributed to the effects of the glycated EHS on Ca^2+^ signaling. Alternatively, the *in vitro* glycation procedure may have resulted in less extensive glycation of fibronectin or generation of glycated species different to those present in EHS (Bishara et al., 2002a). The present study, therefore, involved *in vitro* glycation of bovine serum albumin (BSA) followed by full characterization of the AGE species generated using a variety of techniques including assay of unmodified amine groups, gas chromatograph–mass spectroscopy (GC-MS), and reverse phase high-performance liquid chromatography (HPLC). The effects of exogenously applied AGEs on Ca^2+^ signaling in BAEC were then examined. As many of the deleterious effects of AGEs are mediated by the activation of RAGE and NAD(P)H oxidase (Farmer and Kennedy, 2009; Warboys et al., 2009; Yan et al., 2009), the actions of AGEs on Ca^2+^ signaling were also examined in the presence of NAD(P)H oxidase inhibitors.

## Materials and Methods

### Preparation of glycated protein

Bovine serum albumin (10 or 75 mg/ml in PBS, pH 7.4; 0.15 and 1.13 mM, containing 8.85 or 66.7 mM lysine respectively) was incubated with varying concentrations of either glucose or glucose-6-phosphate (G6P; 5–250 mM, 0.7–28 times excess over lysine) for 5–90 days at 37°C in the dark. The glycation reaction was terminated by dialysis against fresh PBS. Prior to use in experiments, AGE preparations were passed through Detoxi-Gel AffinityPak™ columns to remove bacterial endotoxin. Endotoxin levels in samples before and after column-purification were measured using a modified Limulus Amebocyte Lysate (LAL) test (BioWhittaker, Inc., Walkersville, MD, USA) as per manufacturer’s instructions. Samples used contained <0.01 EU/ml endotoxin (lower limit of reliable detection).

### Assay of glycated protein by fluorescence, absorbance, and assay of unmodified amine groups

The extent of protein glycation was determined by fluorescence (Ex 370 nm/Em 440 nm) and absorbance (340 nm) measurements together with an assessment of unmodified primary amines using fluoraldehyde *o*-phthalaldehyde (OPA) crystals (Thermo Fisher Scientific, Scoresby, VIC, Australia; see Roth, 1971).

### Assay of glycated protein by gas-liquid chromatograph-mass spectroscopy

Gas-liquid chromatograph-mass spectroscopy (GC-MS) analysis of samples was carried as described elsewhere (Dunn et al., 1991; Dyer et al., 1991; Ahmed et al., 1997). Briefly, samples (containing approximately 4 mg protein) were reduced in 100 mM of NaBH_4_ in 0.1 N NaOH for 16 h at 4°C. Excess of NaBH_4_ was discharged by addition of 10% trichloroacetic acid (w/v), samples were then centrifuged at 2000 × *g* and the precipitate resuspended in 0.1 N NaOH. Acid hydrolysis was performed in 6 N HCl and the samples dried *in vacuo* and converted to their trifluoroacetyl methyl ester (TFAME) derivatives for analysis. For preparation of TFAME, 1 ml freshly made 1 M methanolic HCl was added to the samples and incubated for 45 min at 65°C. Solvent was evaporated and the product was redissolved in 1 ml of trifluoroacetic anhydride and the mixture incubated at room temperature for 1 h to obtain the trifluoroacetyl derivatives. After removing the solvent the sample was dissolved in 150 μl of methylene chloride and 2 μl of this solution was injected for GC/MS analysis.

Gas-liquid chromatograph/MS analyses were performed on a Hewlett-Packard Model 5890 GC equipped with a Model 7673A Autosampler and Model 5970 Mass Selective Detector, using a 30-m DB-5 capillary column (Agilent Technologies). The injection port was maintained at 275°C and the transfer line at 290°C. The temperature program was as follows: 3 min at 130°C; ramp to 180°C at 4°/min and then to 240°C at 5°/min and to 290°C at 15°/min; hold for 5 min at 290°C. Quantification was based on isotope dilution using standard curves constructed from mixtures of a constant amount of heavy labeled internal standards (d4CML, d8CEL) and increasing amounts of non-labeled carboxymethyl lysine (CML) and CEL. The limit of detection for GC/MS analysis of CML and CEL was 0.05 and 0.007 mmol/mol of Lys respectively, which is comparable with previous data (Miyata et al., 2000). The coefficient of variation for detection of the analytes was 5% (Dunn et al., 1991). The average CML and CEL recovery was 91% (Petrovic et al., 2005).

### Assay of glycated protein by reverse phase HPLC

Glycated and control BSA samples (2 mg protein) were precipitated and hydrolyzed (Nobecourt et al., 2010), derivatized using *o*-phthaldialdehyde, and subjected to HPLC with fluorescence detection as previously described (Zeng and Davies, 2005). Samples were separated (flow rate 1 ml/min) using a gradient of 85% buffer A (96% 50 mM sodium acetate, pH 6.5, and 4% methanol; v/v) and 15% buffer B (100% methanol) for 40 min; 15–50% buffer B over 58 min; 50–90% buffer B for 2 min; 90% buffer B for 2 min; 90–15% buffer B over 5 min; and re-equilibration at 15% buffer B for 8 min. Identities of peaks were confirmed by elution times and spiking with authentic materials. Peak areas were converted to absolute levels using standard curves constructed using authentic *S*-carboxymethylcysteine (CMC), CML, CEL, (2S)-2-amino-5-(5-methyl-4-oxo-4,5-dihydro-1H-imidazol-2-ylamino)-pentanoic acid (MG-H1), (2S)-2-amino-5-(2-amino-5-methyl-4-oxo-4,5-dihydro-imidazol-1-yl)-pentanoic acid acetate (MG-H2), glyoxal-derived lysine dimer (1,3-bis(5-amino-5-carboxypentyl)-3H-imidazolium acetate; GOLD), and methylglyoxal-derived lysine dimer (1,3-bis(5-amino-5-carboxypentyl)-4-methyl-3H-imidazolium acetate; MOLD). The limit of detection for any AGE using this HPLC method is 0.1 pmol.

### BAEC culture and Ca^2+^ measurements

Bovine aortic endothelial cells (Cambrex Bio Science Australia Pty Ltd.) were cultured in endothelial cell growth medium (BulletKit^®^; Cambrex Bio Science Australia Pty Ltd.) at 37°C in 5% CO_2_ (passages 3–7). Cells were allowed 72 h to adhere and become confluent on sterile, single-well MatTek™ plates (MatTek Corporation, Ashland, MA, USA). Intracellular [Ca^2+^] in BAEC was measured using the fluorescent, ratiometric dye Fura-2 AM. BAEC were washed three times with Krebs solution (composition in mM: 111 NaCl, 25.7 NaHCO_3_, 4.9 KCl, 2.5 CaCl_2_, 1.2 MgSO_4_, 1.2 KH_2_PO_4_, 11.5 glucose, and 10 4-(2-Hydroxyethyl)piperazine-1-ethanesulfonic acid, *N*-(2-Hydroxyethyl)piperazine-*N*′-(2-ethanesulfonic acid; HEPES) dissolved in double distilled water, pH 7.4) and incubated with Fura-2 AM (2 μM) at 37°C for 30 min. After washing, fluorescence measurements were performed in enclosed cover-slip holders perfused with Krebs solution (composition described above) at room temperature on a randomly selected area of 25 cells that were highly confluent with even dye loading. The Krebs solution used to superfuse the cells during fluorescence measurements was not gassed with CO_2_, our experience has shown HEPES is sufficient to buffer the Krebs and any pH changes in the environment of the cells over the brief time-course of the experiment that, including the Fura-2 incubation period, was less than an hour (Bishara et al., 2002a). Fluorescence measurements were made using an Olympus Cell^R^ Real-Time Imaging Station (objective 40×; Olympus Australia Pty Ltd., Mt Waverley, VIC, Australia). Images were collected at one image per 5, 15, or 30 s based on the stage of experiment as shown on each graph. Changes in intracellular [Ca^2+^] were expressed as the ratio of emission (510 nm) following excitation at 340 and 380 nm. Data was normalized to the baseline, which was defined as values obtained during the period (1 min) immediately prior to intervention.

### Experimental protocols

Following Fura-2 loading, the cells were incubated with an AGE preparation or control solution (the same concentration of BSA incubated under identical conditions as the glycated samples, but in the absence of glycating agent) for 5 min prior to addition of a Ca^2+^ signaling agent. Fura-2 fluorescence was monitored from 1 min prior to the addition of the control or AGE sample and for the following 11 min. To delineate effects on intracellular Ca^2+^ release and extracellular Ca^2+^ entry including CCE, BAECs were incubated with Ca^2+^-free Krebs solution (containing no added CaCl_2_) for 1 min prior to addition of the AGE preparation or control solution. Cells were then incubated with the AGE preparation or control solution for 5 min before addition of one of ATP, thapsigargin, or ionomycin. Extracellular Ca^2+^ entry was induced by subsequent addition of buffer containing CaCl_2_ (1 mM; Bishara et al., 2002a), in the continued presence of ATP, thapsigargin, or ionomycin, and fluorescence was recorded for an additional 5 min. Where required, the anti-oxidant dl-α-lipoic acid (2 μM), the NAD(P)H oxidase inhibitors diphenyleneiodonium (DPI, 1 μM) or 4′-Hydroxy-3′-methoxyacetophenone (apocynin, 500 μM) or the IP_3_ receptor antagonist xestospongin C (1 μM) were incubated with BAECs for 30 min at 37°C prior to the addition of AGE or control solutions. Where experiments varied from this protocol, such deviations are described in the text. Appropriate buffer controls were performed for these agents, the buffer controls had no effect on the parameters measured in the present study (not shown).

### Drugs and chemicals

Quick Start™ Bradford assay and sodium dodecyl sulfate (SDS) from Bio-Rad, Phosphate buffer saline tablets (PBS), Fura-2 AM, and trypsin-EDTA from Invitrogen Australia Pty Limited (Mulgrave, VIC, Australia), Detoxi-Gel Affinity Pack™ Pre-Packed columns from Thermo Fisher Scientific Inc. (Mulgrave, VIC, Australia), BSA from BOVOGEN (Essendon, VIC, Australia), LAL QCL-1000 from BioWhittaker Inc., Water for Irrigation from Baxter Healthcare Pty Ltd. (Old Toongabbie, NSW, Australia), Endothelial growth medium (EGM) BulletKit^®^ from Cambrex Bio Science Australia Pty Ltd., CML for HPLC from TRC (ON, Canada), CEL, MG-H1, MG-H2, GOLD, and MOLD from NeoMPS (Strasbourg, France). Unless otherwise stated, all other drugs and chemicals were obtained from Sigma Aldrich Pty Ltd. (Castle Hill, NSW, Australia).

### Data analysis and statistical methods

Overall changes in normalized fluorescence ratio are expressed as AUC (area under curve) primarily because the response to AGEs (as opposed to ATP) did not feature clear “peak” or “sustained” phases, but a slowly developing and sustained increase. Calculation of AUC was performed using GraphPad Prism Version 4.01 (GraphPad Software Inc., La Jolla, CA, USA) with background fluorescence subtracted (from a region of the cover slips not containing cells). “Curves” were defined as the period between which fluorescence ratio significantly exceeded and returned to the basal level following an intervention, in other words elevations in basal fluorescence caused by AGEs were excluded. Overall, fluorescence data for an individual experiment were normalized to the baseline and presented as mean ± SEM. Comparisons of the means and SEMs of two groups were performed using unpaired *t*-tests. Multiple comparisons were performed using two-way ANOVA with Bonferroni post-tests. All other statistical operations were performed using GraphPad Prism 4.01. *n* is the number of plates and represents at least three different batches of plated cells.

## Results

### Characterization of glycated BSA

**Figure 1:**
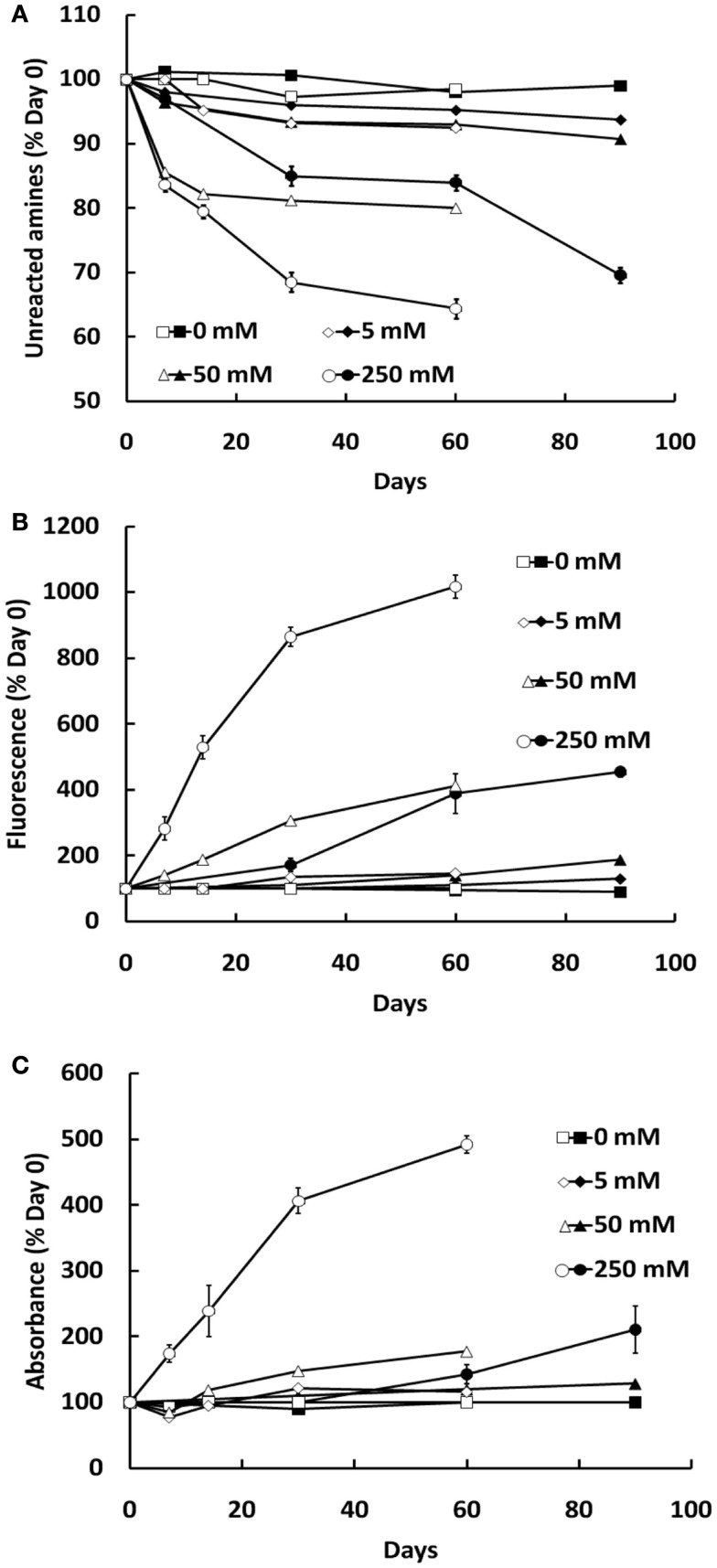
**Glycation of BSA (10 mg/ml) during co-incubation with G6P (open symbols) or glucose (filled symbols), as assessed by the decline in free amine groups (A), sample fluorescence (B), and sample absorbance (C)**. Symbols represent the mean ± SEM for three separate batches of BSA glycated under identical conditions and each sample was read in triplicate.

**Figure 2:**
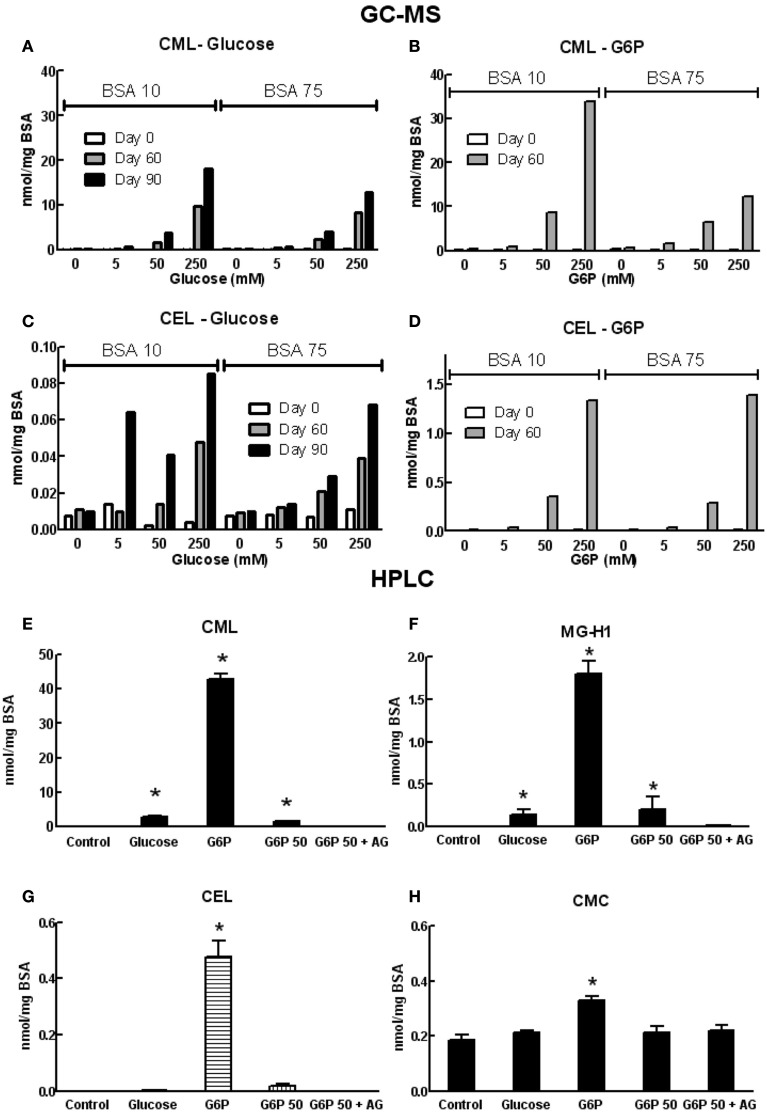
**Concentrations of various AGE in samples of co-incubated BSA and glycating agent analyzed using GC-MS (A–D) or HPLC [(E–H); see list of abbreviations for individual AGE species definitions]**. Columns in **(A–D)** represent a triplicate reading of a single sample; columns in **(E–H)** represent the mean ± SEM for three separate batches of BSA glycated under identical conditions and each sample was read in duplicate. *Indicates significant increase from control (*P* < 0.05, *t*-test).

Formation of AGEs in BSA solutions co-incubated with G6P or glucose was dependent on both the duration of incubation and concentration of glycating agent, as assessed by sample absorbance, fluorescence, assay of unmodified amines (Figure 1) and GC-MS (Figures 2A–D) and HPLC (Figures 2E–H). Analysis showed CML to be the predominant AGE (of those assayed) formed in the BSA samples, regardless of glycating agent (Figure 2). Of the other AGEs, significant increases in CEL, MG-H1, and CMC were also found, when compared with control BSA samples (Figure 2). There was no significant increase in the formation of MG-H2, GOLD, or MOLD (data not shown). G6P proved to be a more effective glycating agent than glucose (Figures 1 and 2).

### Effect of glycated BSA on Ca^2+^ signaling in BAEC

The most extensively glycated BSA sample (10 mg/ml BSA incubated with 250 mM G6P for 60 days), at a final concentration of 0.1 mg protein/ml, was used routinely to examine effects on Ca^2+^ signaling in BAEC. Control samples consisted of 0.1 mg/ml of a 10 mg/ml BSA solution incubated without glycating agent for 60 days.

**Figure 3:**
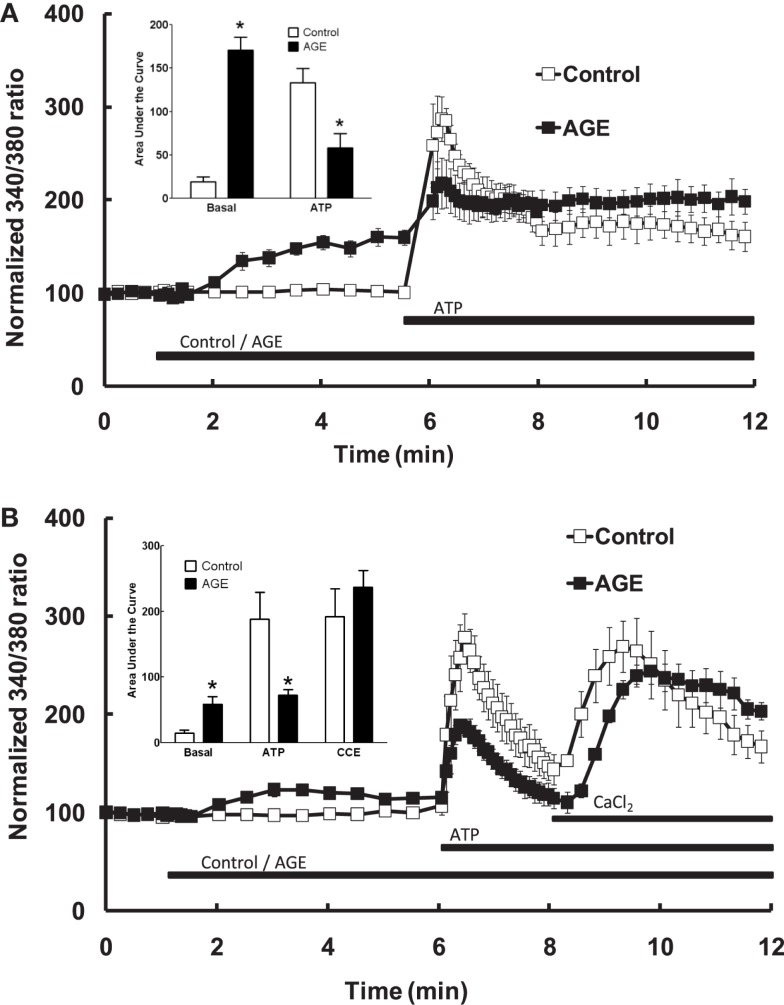
**Effect of AGEs on ATP-induced Ca^2+^ signaling in BAEC**. **(A)** In the presence of extracellular Ca^2+^, AGEs alone induced an increase in [Ca^2+^] and inhibited the subsequent ATP-induced Ca^2+^ increase, as shown in the inset (Area Under Curve data). **(B)** In the absence of extracellular Ca^2+^, AGEs alone induced an increase in [Ca^2+^], inhibited ATP-induced intracellular Ca^2+^ release but did not affect CCE upon the re-addition of extracellular Ca^2+^ (CaCl_2_). Points and columns represent the mean ± SEM of five to six recordings. *Indicates significant effect of AGEs compared to control (*P* < 0.05, *t*-test).

In endothelial cells bathed in normal (i.e., Ca^2+^-replete) Krebs solution, exposure of the endothelial cells to the AGE sample alone caused a significant increase in intracellular [Ca^2+^], whereas the control sample did not increase [Ca^2+^] (Figure 3A). The difference in Ca^2+^ response was evident approximately 1 min. following addition of the sample and reached a plateau after approximately 3 min. exposure (Figure 3A). In cells incubated with control sample, subsequent exposure to ATP (100 μM) caused a rapid increase in intracellular [Ca^2+^] (compared with the AGEs-induced increase) consisting of an early, transient increase peaking at approximately threefold the normalized basal fluorescence ratio, followed by a sustained increase in fluorescence ratio at about 1.5 times the basal level (Figure 3A). Exposure of cells to the AGE-containing sample significantly attenuated the ATP-induced increase in [Ca^2+^] (compared to the pre-ATP, “baseline” level) with an apparently selective effect on the early, transient component (Figure 3A).

**Figure 4:**
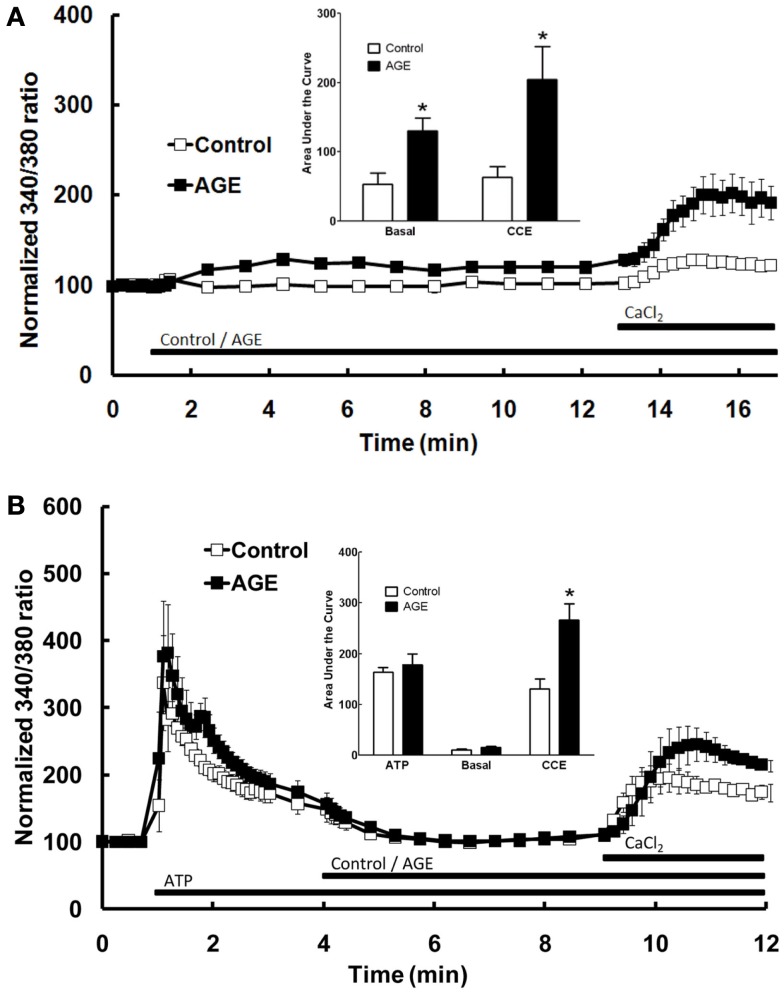
**Effect of AGEs on Ca^2+^ signaling in BAEC**. **(A)** In the absence of extracellular Ca^2+^, AGEs alone induced an increase in [Ca^2+^] and induced significant CCE compared with control (unglycated BSA). **(B)** In the absence of extracellular Ca^2+^, AGEs added to the BAEC after ATP did not induce an increase in [Ca^2+^], but increased CCE (CaCl_2_). Points and columns represent the mean ± SEM of six recordings. *Indicates significant effect of AGEs compared to control (*P* < 0.05, *t*-test).

The effect of AGEs on the different components of Ca^2+^ signaling were investigated further by superfusing the cells with Ca^2+^-free and Ca^2+^-replete buffer solutions. In the absence of extracellular Ca^2+^, all increases in intracellular [Ca^2+^] are due to the release of Ca^2+^ from intracellular stores only. Under these conditions, AGEs alone caused a sustained increase in intracellular [Ca^2+^] (Figure 3B), although the [Ca^2+^] increase was significantly less than that observed in Ca^2+^-containing buffer. In the absence of extracellular Ca^2+^, AGEs inhibited the following ATP-induced release of intracellular Ca^2+^ (Figure 3B). In contrast, the increase in [Ca^2+^] following re-introduction of Ca^2+^-containing buffer was not significantly altered by the glycated protein (Figure 3B), suggesting a specific effect on the Ca^2+^-release component. Further, re-addition of Ca^2+^-replete buffer to the cells in the presence of AGEs only (i.e., in the absence of ATP) showed that AGEs alone could induce extracellular Ca^2+^ entry (Figure 4A). When the order of AGEs and ATP application to the cells was reversed (that is, AGEs were added following ATP), AGEs did not cause an increase in intracellular Ca^2+^ (Figure 4B), suggesting AGEs and ATP released Ca^2+^ from the same intracellular “pool.” Under this protocol, AGEs enhanced subsequent Ca^2+^ entry caused by the re-addition of Ca^2+^-containing buffer to the cells (Figure 4B).

**Figure 5:**
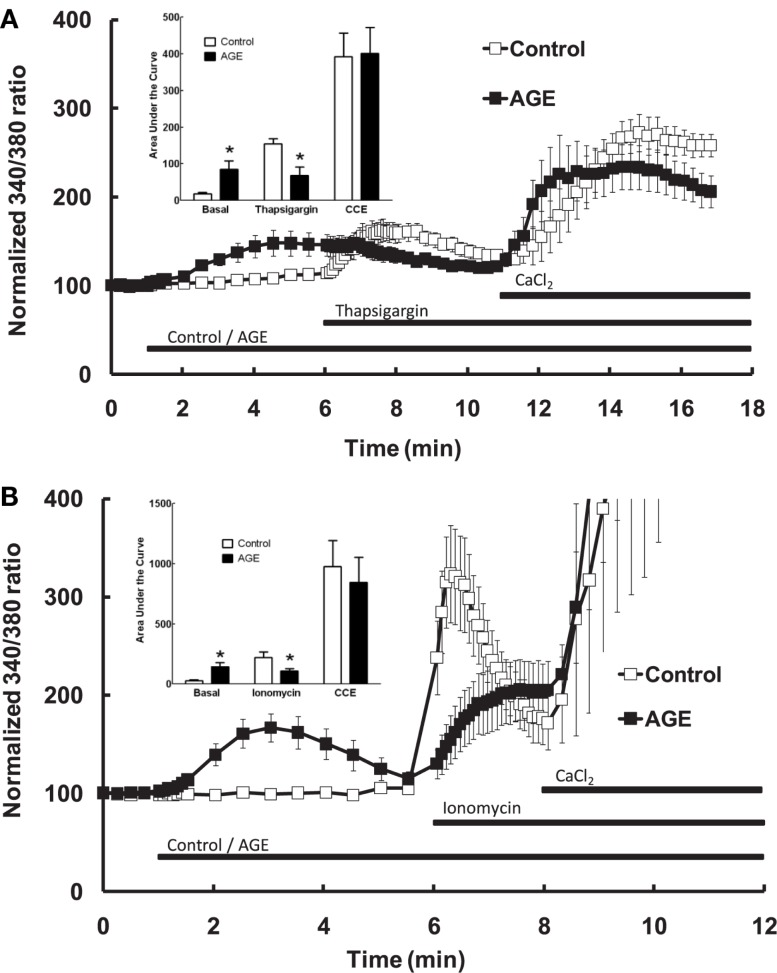
**Effect of AGEs on thapsigargin (A) and ionomycin (B)-induced Ca^2+^ signaling in BAEC, in the absence of extracellular Ca^2+^**. In both cases AGEs alone induced an increase in [Ca^2+^] and inhibited the subsequent agent-induced Ca^2+^ increase, but did not affect CCE upon the re-addition of extracellular Ca^2+^ (CaCl_2_). Points and columns represent the mean ± SEM of six recordings. *Indicates significant effect of AGEs compared to control (*P* < 0.05, *t*-test).

Additional experiments were subsequently performed to examine the effects of AGEs on endothelial cell intracellular Ca^2+^ release induced by thapsigargin, an inhibitor of endoplasmic reticulum Ca^2+^-ATPase pump which causes emptying of intracellular Ca^2+^ stores, and ionomycin which acts directly on the intracellular Ca^2+^ storage organelle to empty the store. In the absence of extracellular Ca^2+^, AGEs inhibited intracellular Ca^2+^ release in response to either thapsigargin (0.1 μM; Figure 5A) or ionomycin (3 μM; Figure 5B). Under both of these protocols, AGEs again did not affect Ca^2+^ entry induced by the re-addition of Ca^2+^-containing buffer to the cells; in this case, such entry may be termed capacitive Ca^2+^ entry or CCE, as these agents have not been shown to activate other Ca^2+^-entry mechanisms in vascular endothelial cells. The time-course of the thapsigargin experiments was extended slightly due to the prolonged time over which thapsigargin stimulated Ca^2+^ release (Figure 5A). Collectively, these data support a specific effect of AGEs on Ca^2+^ release from the endoplasmic reticulum, consistent with partial depletion of the intracellular Ca^2+^ store.

### Effects of the extent of glycation and AGE concentration on Ca^2+^ signaling

**Figure 6:**
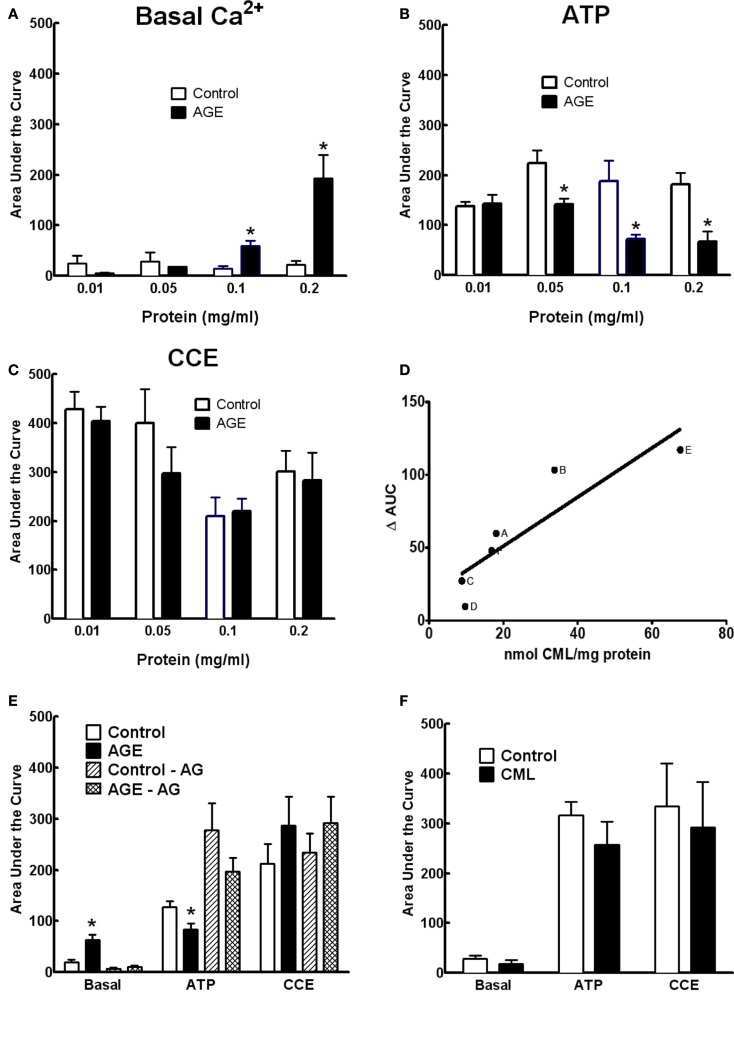
**Concentration dependence of AGE effects on Ca^2+^ signaling in BAEC (A–C)**. **(D)** Correlation between CML content and inhibition of ATP-induced Ca^2+^ increase in BAEC of various AGE samples [**(A)**, 250 mM glucose/90 days; **(B)**, 250 mM G6P/60 days; **(C)**, 50 mM G6P/60 days; **(D)**, 250 mM glucose/60 days; **(E)**, 0.2 mg/ml of 250 mM G6P/60 days; **(F)**, 0.05 mg/ml of 250 mM G6P/60 days]. **(E)** AGE sample (50 mM G6P/60 days) co-incubated with aminoguanidine (AG, 25 mM) did not alter Ca^2+^ signaling, compared with AGE not containing AG. **(F)** CML (6 μM) did not alter Ca^2+^ signaling in BAEC. Points and columns represent the mean ± SEM of five to six recordings. *Indicates significant effect of AGE compared to control (*P* < 0.05, *t*-test).

The effects of AGEs on Ca^2+^ signaling were dependent upon both the concentration of glycated BSA and the extent of glycation. Figures 6A–C show the effects of the AGEs sample on basal [Ca^2+^] and ATP-induced Ca^2+^ release were concentration dependent; concentrations of this sample exceeding 0.2 mg/ml were shown to be detrimental to cell viability as determined by propidium iodide staining (not shown). In samples of BSA incubated under varying conditions (concentration, time of exposure, and type of glycating agent), there was a strong correlation between the concentration of CML, taken as indicator of the extent of glycation, and inhibition of ATP-induced Ca^2+^ increase in BAEC (Figure 6D). The inhibitor of advanced glycation aminoguanidine (25 mM), co-incubated with the sample 50 mM G6P/10 mg/ml BSA/60 days, not only prevented the accumulation of CML and MG-H1 in this sample (see Figure 2) but also prevented any effect on Ca^2+^ signaling (Figure 6E).

The concentration of CML in 0.1 mg/ml of the AGE sample was estimated to be approximately 0.6 μM. Ten-fold of this concentration (6 μM) of CML alone, as opposed to CML adducts formed on glycated BSA, had no effect on Ca^2+^ signaling in BAEC (Figure 6F), suggesting that while CML levels correlate with extent of glycation they may not, themselves, directly impair Ca^2+^ signaling.

### Effect of anti-oxidant and NAD(P)H oxidase inhibitors on AGE and Ca^2+^ signaling

**Figure 7:**
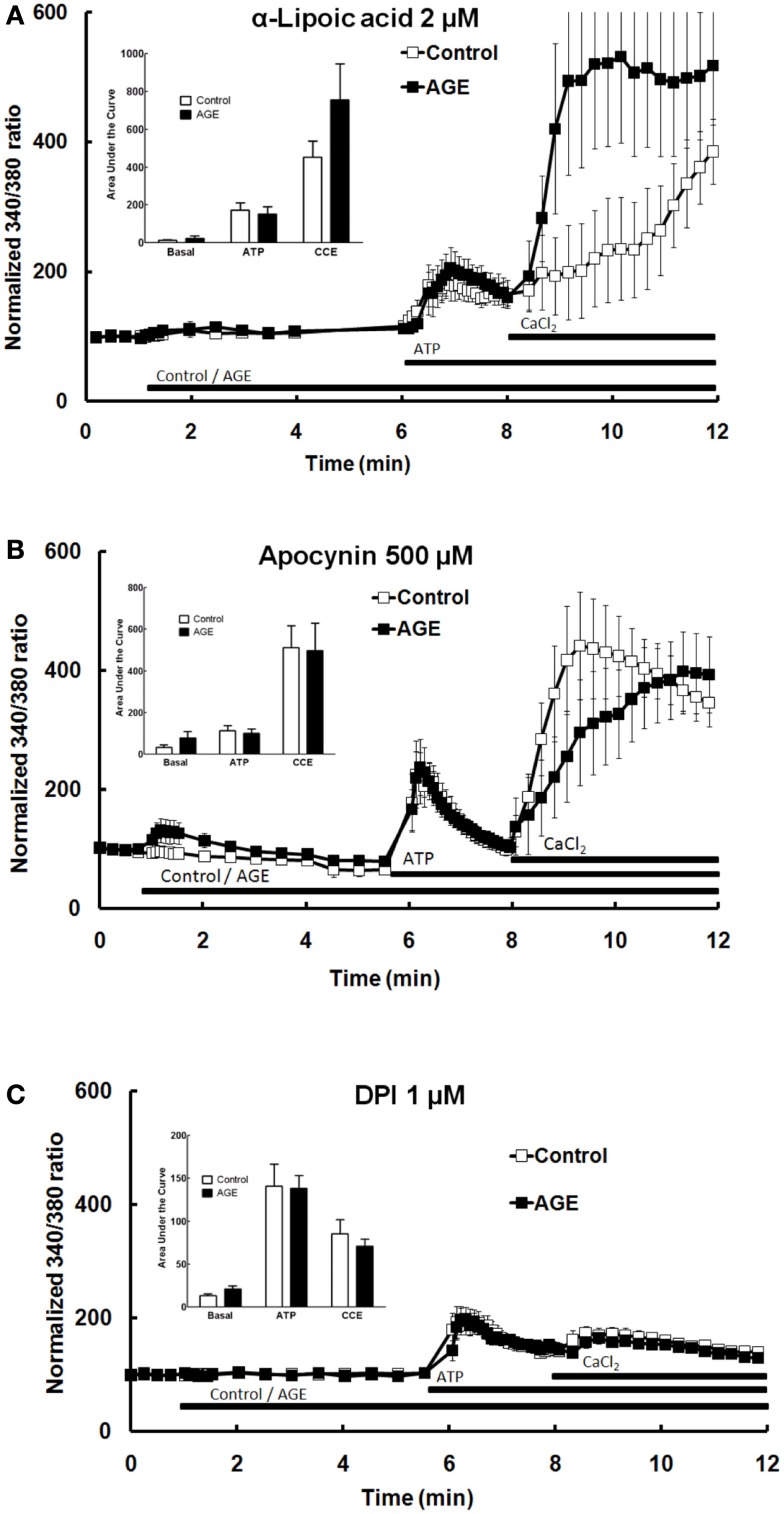
**α-Lipoic acid (A), apocynin (B), and DPI (C) each abolished the effects of AGEs on Ca^2+^ signaling in BAEC**. Points and columns represent the mean ± SEM of 6–11 recordings.

A number of previous studies have suggested that the effects of AGE are mediated by ROS, subsequent to activation of the enzyme NAD(P)H oxidase (Farmer and Kennedy, 2009; Warboys et al., 2009). The role of ROS and NAD(P)H oxidase in the effects of AGEs on Ca^2+^ signaling in BAEC were examined using the anti-oxidant α-lipoic acid (2 μM) and the NAD(P)H oxidase inhibitors apocynin (500 μM) and DPI (1 μM). Pre-incubation of the cells with each of these compounds prevented both the AGEs-induced release of intracellular Ca^2+^ and the inhibition of ATP-mediated Ca^2+^ release caused by AGEs (Figures 7A–C). DPI alone significantly inhibited CCE (Figure 7C). DPI and, to a lesser extent, lipoic acid inhibited both components of ATP-induced Ca^2+^ signaling (Figure 7).

### Role of IP_3_ in effects on AGE-induced changes in Ca^2+^ signaling

**Figure 8:**
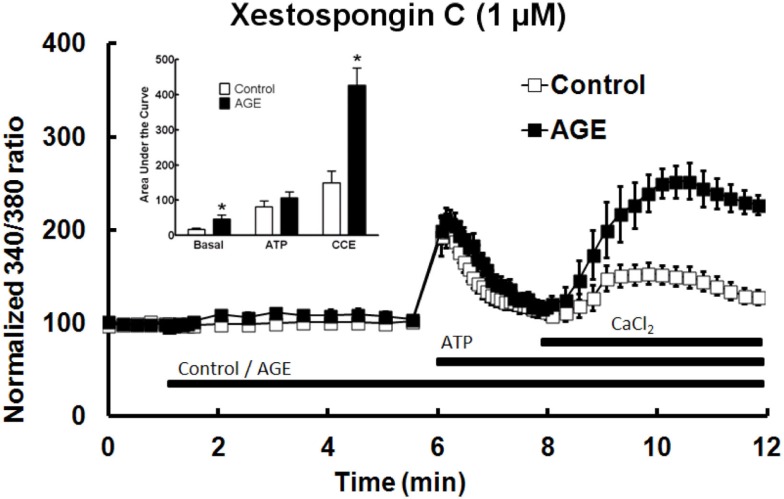
**Xestospongin C attenuated the effects of AGEs on basal [Ca^2+^] and abolished the effects on ATP-induced Ca^2+^ release, however AGEs, in the presence of xestospongin C, enhanced CCE**. Columns represent the mean ± SEM of six recordings. *Indicates significant effect of AGEs compared to control (*P* < 0.05, *t*-test).

The role of IP_3_ in the effects of AGEs on Ca^2+^ signaling in BAEC was investigated using the IP_3_ receptor antagonist xestospongin C (1 μM). In the presence of the IP_3_ blocker, AGEs significantly increased basal [Ca^2+^] in the cells, but did not inhibit ATP-induced Ca^2+^ release (Figure 8). The combination of AGEs and xestospongin C significantly increased extracellular Ca^2+^ entry compared with Ca^2+^ entry in the presence of the control sample and xestospongin C (Figure 8). Xestospongin C did not interfere with recording by fluorescing at the same wavelength as Fura (data not shown).

## Discussion

Dysfunction of the vascular endothelium is an early and potentially damaging vascular complication of diabetes which may contribute to the vascular complications associated with diabetes. AGEs formed following prolonged exposure of plasma and matrix proteins and other macromolecules to increased blood glucose are known to cause endothelial dysfunction (Wautier and Schmidt, 2004; Goldin et al., 2006). As many of the functions of vascular endothelial cells depend upon Ca^2+^ signaling, this study examined the effects of acute exposure to AGEs on Ca^2+^ signaling in endothelial cells. Incubation of the cells with AGEs caused an acute depletion of intracellular Ca^2+^ stores, the emptying of these stores resulting in an AGE-induced increase in cell [Ca^2+^]. The initial increase in endothelial cell [Ca^2+^] caused by agonists such as ATP, along with agents including ionomycin and thapsigargin, is also due to the release of these stores; prior incubation with AGE depletes these stores and therefore less releasable Ca^2+^ is available for the other agents (e.g., ATP), thus ATP-induced [Ca^2+^] release was inhibited during this period. The sustained phase of the ATP-induced [Ca^2+^] increase is due to influx of extracellular Ca^2+^ and, as established by subsequent experiments, this component of the endothelial cell [Ca^2+^] increase does not appear to be altered by AGE. This is possibly because AGEs alone induced CCE or Ca^2+^ entry through activation of intracellular Ca^2+^ stores, thus complicating such observations. These effects of AGEs on Ca^2+^ signaling may be mediated by superoxide or its derivatives as they were prevented by inhibitors of NAD(P)H oxidase.

Advanced glycation end products were formed by incubation of BSA with glucose or G6P and the individual AGE adducts generated were quantified using GC-MS and HPLC. G6P proved a more effective glycating agent than glucose, as others have observed previously (Bierhaus et al., 1998; Singh et al., 2001; Valencia et al., 2004) and CML was by far the most abundant adduct formed, exceeding that of the next most abundant AGEs assayed (CEL and MG-H1) by at least 20-fold. Previous studies have shown the plasma concentration of CML correlates with the severity of diabetic complications such as retinopathy and nephropathy (Reddy et al., 1995; Furth, 1997; Wagner et al., 2001). Furthermore, in adults with diabetes as well as adolescents and children with Type 1 diabetes, serum CML levels were significantly elevated, particularly in those with microvascular complications and the plasma concentration of CML in these patients similar to that in the *in vitro* glycated samples generated in the present study (Berg et al., 1998; Hwang et al., 2005; Alkhalaf et al., 2012). Concentrations of the dominant AGE product (CML) in non-diabetic adults are reported between 0.6 and 1.76 mmol/mol lysine, increasing up to 1.33–2.37 mmol/mol lysine in Type 1 diabetic subjects with microvascular complications (Dyer et al., 1993; McCance et al., 1993), varying dependent upon the age of the subjects, vascular complication status, and the analytical methods used for measurement (Dyer et al., 1993; McCance et al., 1993; Semba et al., 2010; Alkhalaf et al., 2012). In the current study, the final concentration of CML in the “standard” AGE used (0.1 mg/ml of 250 mM G6P and 10 mg/ml BSA, 60 days) was 3.3–4.0 mmol/mol lysine, similar in magnitude to those concentrations reported in diabetic patients. Several studies have demonstrated significant glycation of albumin in various cohorts of diabetic patients, with the percentage of glycation similar to or exceeding that of the most heavily glycated sample utilized in the present study as assessed by fluorescence, absorbance, and unreacted amine measurements (about 35% glycation; Rondeau and Bourdon, 2010; Kim and Lee, 2012). It would therefore be reasonable to assume that the glycation reactions used in the present study yielded physiologically relevant concentrations of glycated albumin and CML and provided a valid basis for studying the effects of AGEs *in vitro*.

Several observations support the idea that AGEs inhibited Ca^2+^ signaling in the endothelial cells by causing a relative depletion of the intracellular Ca^2+^ stores. AGEs alone caused an increase in intracellular [Ca^2+^], both in the presence and absence of extracellular Ca^2+^. In the absence of extracellular Ca^2+^, AGEs inhibited the release of intracellular [Ca^2+^] caused by three different mechanisms; IP_3_-mediated release (using ATP; see also Lynch et al., 1992; Bishara et al., 2002b; Kwan et al., 2009), prevention of Ca^2+^ uptake into the stores through inhibition of Ca^2+^-ATPase (thapsigargin) and by the Ca^2+^ ionophore ionomycin, which acts directly upon the storage organelle membrane (Kauffman et al., 1980; Bolger et al., 1983). AGEs alone could induce extracellular Ca^2+^ entry (Figure 4A), most likely CCE as this is a phenomenon reliant upon depletion of intracellular Ca^2+^ stores (in particular the endoplasmic reticulum; Parekh, 2006) and AGEs failed to increase intracellular [Ca^2+^] when added to the cells following store depletion using ATP. These observations suggested the AGE-releasable pool of intracellular Ca^2+^ was at least the same size as, if not a subset of, the ATP (i.e., IP_3_)-releasable pool. Collectively, these observations suggest that AGEs inhibited ATP-, thapsigargin-, and ionomycin-induced intracellular Ca^2+^ release by depleting the pool of available Ca^2+^ acted upon by these agents. CCE was not affected by AGEs and, when AGEs were added to the cells following ATP, or when IP_3_ receptors were blocked, extracellular Ca^2+^ entry was enhanced, suggesting the glycated proteins may have an action to induce Ca^2+^ entry or enhance CCE into the BAEC. It should be noted that, unlike thapsigargin and ionomycin, ATP-induced influx of extracellular Ca^2+^ into vascular endothelial cells probably occurs by mechanisms additional to CCE, mostly likely receptor-operated channels such as TRPC1, 3, and 4 (Kamouchi et al., 1999; Brough et al., 2001; Freichel et al., 2001; Sandow et al., 2012), along with other possible channels (Kwan et al., 2009) and membrane Ca^2+^-exchangers such as Na^+^-Ca^2+^ (Berra-Romani et al., 2010). The mechanism of CCE into vascular endothelial cells is not fully understood and somewhat controversial (Beech, 2009) with various TRP channels identified as being involved in the process (Cioffi et al., 2005; Antoniotti et al., 2006; Senadheera et al., 2012; Sonkusare et al., 2012) along with the proteins crucial for I_CRAC_, Stim1, and Orai1 (Abdullaev et al., 2008; Hirano et al., 2009; Antigny et al., 2011). The mechanism of CCE was not addressed in the present study, but the fundamental point is that acute exposure to AGEs did not inhibit extracellular Ca^2+^ entry following store depletion and re-addition of Ca^2+^, under several different experimental, and mechanistically distinct, conditions.

The current study supports the conclusions of Bishara et al. (2002a), in which BAEC or rat heart endothelial cells were co-cultured with glycated matrix proteins (EHS) overnight (16 h). These cells showed elevated basal [Ca^2+^] and impaired ATP-, bradykinin-, and thapsigargin-induced Ca^2+^ signaling, which was suggested to be due to depletion of intracellular Ca^2+^. The co-culture procedure had a significant, negative effect on cell viability, and the present study utilized AGE concentrations that did not alter BAEC viability. AGEs also inhibited both intracellular [Ca^2+^] release and CCE in human mesangial cells following 60 min exposure (Mene et al., 1999), suggesting effects of AGEs on CCE may develop with chronic, rather than acute exposure. AGEs-induced apoptosis of BAEC was also associated with increased [Ca^2+^], an effect also observed in the absence of extracellular Ca^2+^ (Xiang et al., 2006). Oxidative stress was observed to contribute to both increased Ca^2+^ and apoptosis in these studies (Xiang et al., 2006; Hung et al., 2010). Prolonged exposure to AGEs (96 h) increased intracellular [Ca^2+^] and enhanced sphingosine-1-induced Ca^2+^ responses in porcine coronary artery smooth muscle cells (David et al., 2008), which the authors concluded was due to enhanced activity of ryanodine receptors and Ca^2+^-induced Ca^2+^ release. In mouse cardiac myocytes, over-expression of RAGE decreased cytosolic Ca^2+^, but exposure to AGEs for 24 h prolonged excitation-induced Ca^2+^ transients (Petrova et al., 2002). These effects may be explained by AGE-induced depletion of intracellular Ca^2+^ and induction of Ca^2+^ influx respectively.

The role of AGEs in the effects on Ca^2+^ signaling, rather than other potential protein modifications caused by the co-incubation of BSA and d-glucose or G6P (e.g., oxidation or changes in tertiary structure (Coussons et al., 1997) was implicated by the concentration dependent effects of the glycated proteins and the strong correlation between inhibition of ATP-induced Ca^2+^ release and the extent of BSA glycation as measured by CML concentration. Furthermore, preparations incubated with the glycation inhibitor aminoguanidine, which prevented AGE formation, had no effect on Ca^2+^ signaling in the BAECs. CML, the most abundant AGE in the samples, did not alter Ca^2+^ signaling. This suggests that CML-adducted BSA, rather than the CML itself, induced the Ca^2+^-release and store depletion observed. Alternatively, it is possible another AGE besides CML produced the observed effects on Ca^2+^. For example, arginine-AGEs such as MG-H1 inhibit NOS and other l-arginine-metabolizing and transporting enzymes present in the vascular endothelium, although the concentrations required are in excess of 1 mM (Brouwers et al., 2008; Lai et al., 2010). The role of RAGE in the observed effects of AGE was not examined directly. The AGE receptor is known to exist constitutively in some endothelial cell lines including BAEC (Basta et al., 2002; Marx et al., 2004). It is likely that a receptor is involved in the effects of the AGE, given the reasonably rapid increase in [Ca^2+^] following exposure. The role of RAGE in the effects observed in the present study requires further investigation.

Advanced glycation end products have been shown to stimulate ROS accumulation in vascular endothelial cells (Bierhaus et al., 1997), including BAEC (Kislinger et al., 1999; Wautier et al., 2001; Bishara et al., 2002a). ROS are thought to mediate many of the deleterious effects of AGEs on vascular endothelial cells, including decreased adhesion (Schmidt et al., 1995), increased permeability (Warboys et al., 2009) and apoptosis (Xiang et al., 2006; Hung et al., 2010) in addition to impaired NO production and endothelium-dependent vasodilation. The results of the present study suggest that the AGE-induced depletion of intracellular Ca^2+^ was mediated by ROS derived from NAD(P)H oxidase as the general anti-oxidant α-lipoic acid and NAD(P)H oxidase inhibitors apocynin and DPI abolished the effects of AGE on Ca^2+^ signaling. Neither of these inhibitors is selective; DPI also inhibits NOS and flavin oxidase while apocynin has general anti-oxidant properties (Hancock and Jones, 1987; Stuehr et al., 1991; Heumuller et al., 2008; Selemidis et al., 2008) but it has not been established that either eNOS or flavin-containing enzymes other than NAD(P)H oxidase are involved in Ca^2+^ signaling in these cells. It is worth noting that DPI in particular greatly inhibited the ATP-induced Ca^2+^ response of the cells although the relation between this observation and the known actions of DPI remains to be elucidated. Other effects of AGEs or RAGE activation (mostly on gene expression) involve the activation of NAD(P)H oxidase, and are preventable by apocynin and other inhibitors of this enzyme (Guo et al., 2008; Coughlan et al., 2009; Farmer and Kennedy, 2009; Warboys et al., 2009). A recent study showed a role for AGE-induced NAD(P)H oxidase activity in the inhibition of endothelium-dependent vasodilation of coronary arterioles from diabetic mice (Gao et al., 2008) and AGE-induced increases in microvascular permeability in the retina (Warboys et al., 2009). These more “acute” responses are similar in time-course to the increase in intracellular Ca^2+^ shown in the present study. Glycated EHS increased the formation of H_2_O_2_ in BAEC (Bishara et al., 2002a) but, using similar fluorescence detection methods, acute exposure to AGEs did not cause a measureable increase in H_2_O_2_ generation in the present study (results not shown).

Previous work utilizing BAEC associated the effects of glycated matrix proteins on Ca^2+^ signaling with increased intracellular [IP_3_] (Bishara et al., 2002a). In the present study the IP_3_ receptor antagonist xestospongin C attenuated the AGE-induced increase in intracellular Ca^2+^ and abolished the inhibitory effect of AGEs on ATP-induced Ca^2+^ release, suggesting the IP_3_ receptor was involved in the effects of AGEs. AGE and RAGE have been shown to activate PLC (Warboys et al., 2009; You et al., 2010); indeed, PLC activation by RAGE may be a necessary step in the subsequent activation of NAD(P)H oxidase (Gao and Mann, 2009; Warboys et al., 2009). This sequence of events would not implicate a role for ROS in the Ca^2+^-releasing effects of AGEs. Alternatively, H_2_O_2_ was shown to directly activate the IP_3_ receptor on the endoplasmic reticulum in HUVEC, inducing Ca^2+^ release (Zheng and Shen, 2005) and similar findings have been made in other cell types (Gonzalez et al., 2006; Takahashi et al., 2007; Gerich et al., 2009). Other studies have suggested NAD(P)H oxidase derived O_2_ can increase the sensitivity of the IP_3_ receptor in endothelial cells (Hu et al., 2000; Madesh et al., 2005).

In summary, AGEs can act acutely to release intracellular Ca^2+^ in BAEC and induce Ca^2+^ influx, most likely CCE, in vascular endothelial cells and this action may thus inhibit Ca^2+^ signaling by physiological agents such as ATP, which also act through these mechanisms. Acute exposure to AGE does not impair CCE and extracellular Ca^2+^ entry and may in fact enhance it. These actions of AGE appear to be mediated by ROS, possibly generated from NAD(P)H oxidase. There may be a role for IP_3_ receptors in these actions, although this remains to be established. Clearly such rapid and significant effects on Ca^2+^ signaling in vascular endothelial cells may play a significant role in the effects of AGEs and diabetes on the Ca^2+^-dependent processes in these cells, including those which impact the regulation of vascular tone.

## Conflict of Interest Statement

The authors declare that the research was conducted in the absence of any commercial or financial relationships that could be construed as a potential conflict of interest.

## Figures and Tables

**Figure A1:**
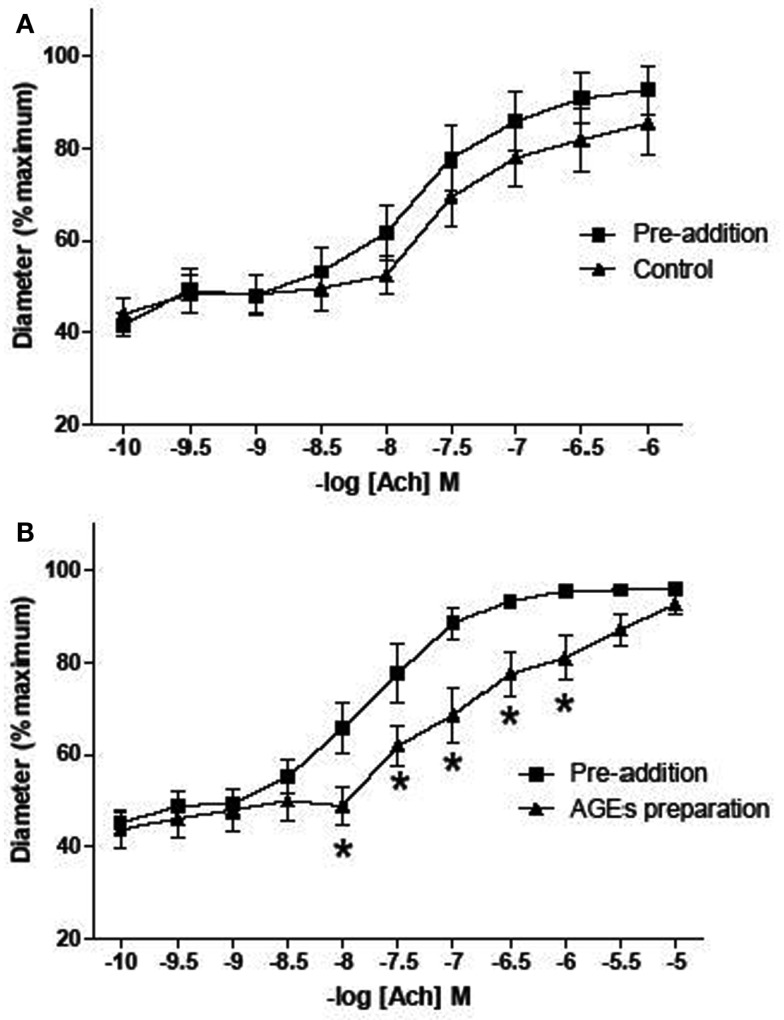
**Effect of AGEs on ACh-induced vasodilatation of arterioles from rat cremaster muscles**. Arterioles were cannulated and maintained at 70 mmHg. **(B)** Five minutes exposure to AGEs inhibited ACh-induced vasodilatation (*n* = 9; *p*-value 0.011, two-way ANOVA). **(A)** Arterioles infused with control AGEs solution did not affect ACh-induced vasodilatation (*n* = 9; *p*-value > 0.05, two-way ANOVA). AGEs were produced by exposing BSA (10 mg/ml) to 250 mM G6P for 60 days. For control AGEs solution, BSA (10 mg/ml) exposed to 0 mM G6P was used. Data are expressed relative to the maximum diameter obtained under passive conditions (in the absence of extracellular calcium) at 70 mmHg. Points represented mean ± SEM.
